# Inflammatory cytokine signalling in vulvovaginal candidiasis: a hot mess driving immunopathology

**DOI:** 10.1093/oxfimm/iqae010

**Published:** 2024-08-17

**Authors:** Kar On Cheng, Dolly E Montaño, Teresa Zelante, Axel Dietschmann, Mark S Gresnigt

**Affiliations:** Junior Research Group Adaptive Pathogenicity Strategies, Leibniz Institute for Natural Product Research and Infection Biology, Hans Knöll Institute (Leibniz-HKI), Beutenbergstraße 11a, Jena, 07749, Germany; Junior Research Group Adaptive Pathogenicity Strategies, Leibniz Institute for Natural Product Research and Infection Biology, Hans Knöll Institute (Leibniz-HKI), Beutenbergstraße 11a, Jena, 07749, Germany; Department of Medicine and Surgery, University of Perugia, Piazza Lucio Severi 1, Perugia, 06132, Italy; Junior Research Group Adaptive Pathogenicity Strategies, Leibniz Institute for Natural Product Research and Infection Biology, Hans Knöll Institute (Leibniz-HKI), Beutenbergstraße 11a, Jena, 07749, Germany; Junior Research Group Adaptive Pathogenicity Strategies, Leibniz Institute for Natural Product Research and Infection Biology, Hans Knöll Institute (Leibniz-HKI), Beutenbergstraße 11a, Jena, 07749, Germany

**Keywords:** *Candida albicans*, vaginal thrush, chemokines, neutrophil recruitment and activation, inflammasome, epithelial activation threshold

## Abstract

Protective immunity to opportunistic fungal infections consists of tightly regulated innate and adaptive immune responses that clear the infection. Immune responses to infections of the vaginal mucosa by *Candida* species are, however, an exception. In the case of vulvovaginal candidiasis (VVC), the inflammatory response is associated with symptomatic disease, rather than that it results in pathogen clearance. As such VVC can be considered an inflammatory disease, which is a significant public health problem due to its predominance as a female-specific fungal infection. Particularly, women with recurrent VVC (RVVC) suffer from a significant negative impact on their quality of life and mental health. Knowledge of the inflammatory pathogenesis of (R)VVC may guide more effective diagnostic and therapeutic options to improve the quality of life of women with (R)VVC. Here, we review the immunopathogenesis of (R)VVC describing several elements that induce an inflammatory arson, starting with the activation threshold established by vaginal epithelial cells that prevent unnecessary ignition of inflammatory responses, epithelial and inflammasome-dependent immune responses. These inflammatory responses will drive neutrophil recruitment and dysfunctional neutrophil-mediated inflammation. We also review the, sometimes controversial, findings on the involvement of adaptive and systemic responses. Finally, we provide future perspectives on the potential of some unexplored cytokine axes and discuss whether VVC needs to be subdivided into subgroups to improve diagnosis and treatment.

## Introduction

Vulvovaginal candidiasis (VVC), also called vaginal thrush, is a significant public health issue due to its high prevalence as a female-specific infection [1, 2]. VVC is one of the most common vaginal infections worldwide and a principal cause of vaginal and vulvar inflammation [3, 4]. Approximately 75% of women globally suffer from this infection at least once during their reproductive years, and about 7–9% of women experience recurrent VVC (RVVC, at least four episodes annually) [3–7]. VVC, and particularly RVVC, significantly impacts the quality of life and mental health of millions of women [8, 9]. Despite the severe discomfort, including curdy vaginal discharge, itching, pain, burning, redness, and swelling as well as associated medical costs, there are no highly effective diagnostic and therapeutic strategies for (R)VVC [10]. RVVC treatment primarily relies on fluconazole maintenance therapy to prevent reinfection [1, 5], which can drive the occurrence of resistance. In many cases it is difficult to accurately diagnose infection and confirm that the isolated *Candida* species are truly the cause of the symptoms, making it challenging to identify the most effective treatment strategy. The rate of non-*albicans Candida* (NAC) species and the atypical VVC they cause is increasing, which complicates the use of standard treatments [11, 12].

VVC pathogenesis entails a complex combination of fungal commensalism and pathogenicity with simultaneous protective and dysregulated inflammatory responses [13]. Multiple factors can contribute to the onset of VVC, for instance, vaginal microbiome dysbiosis, diabetes, pregnancy, hormone replacement therapy, and the use of oral contraceptives [3]. Nevertheless, these factors are not shared by all VVC or RVVC patients; many women do not exhibit predisposing conditions [1, 5]. VVC sets itself apart from other *Candida* infections as most VVC patients do not have a compromised immune system [14, 15]. In fact, specific immune pathways that mediate protective immunity during systemic and oral candidiasis, appear to contribute to the pathogenesis and severity of VVC. This was suggested by a seminal study, in which women were challenged with live *C. albicans* and exhibited VVC symptoms associated with neutrophil influx rather than the fungal burden alone [16]. In this regard, many further translational research and clinical studies suggest that symptomatic VVC arises from mucosal damage caused by vaginal *Candida* overgrowth inducing dysfunctional neutrophil infiltration [13, 17–20].

Most of the knowledge on VVC pathogenesis has been elucidated using VVC mouse models. While these models helped to acquire valuable insights into disease pathogenesis, mice do not display classic VVC symptoms and exhibit inter-species differences in microbiota, vaginal pH, and the immune system. Moreover, these models do not exhibit recurrence. It has, therefore, been challenging for the field to translate how inflammatory responses and symptomatology are connected in VVC patients. For this reason, patient-focussed studies are crucial to unravel the inflammatory pathogenesis of human VVC.

**Figure 1.**
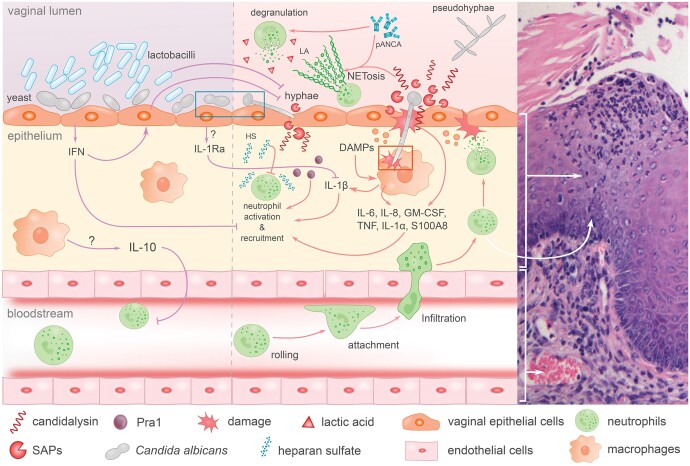
Overview of the pathways preventing and driving immunopathology associated with Vulvovaginal candidiasis. Vaginal epithelial cells promoting a tolerogenic response to *C. albicans* yeast (left). Lactobacilli of the resident microbiota antagonize the pathogenesis of *Candida* species. Epithelial type-I interferon (IFN) responses increase epithelial resistance to infection and dampen neutrophil activation. Potent anti-inflammatory cytokine pathways may play a role in preventing disease-driving inflammatory responses. Epithelial cells have the capacity to mount a strong release of interleukin-1 receptor antagonist (IL-1Ra) that competitively antagonizes signalling by the hallmark VVC cytokine interleukin (IL)-1β. IL-10 is a potent anti-inflammatory cytokine that can be released by various immune cell types to dampen inflammation and maintain homeostasis. Following a shift towards pathogenicity, hyphae-associated virulence factors, such as the antigen Protein 1 (Pra1), candidalysin, and the secreted aspartyl proteases (SAPs), instigate several pro-inflammatory responses (middle). *C. albicans* hyphae invade the mucosa and cause tissue damage, eliciting proinflammatory responses by release of damage-associated molecular patterns DAMPs. NLRP3 inflammasome-processed IL-1β is a key cytokine driving the inflammatory response during VVC, facilitating neutrophil recruitment and the related hyperinflammation. Recruited neutrophils fail to accurately deploy their effector mechanisms due to various vaginal niche specific factors (e.g. perinuclear anti-neutrophil cytoplasmic antibodies, pANCA; and heparan sulphate, HS). The large number of activated neutrophils contributes to collateral tissue damage driving non-self-limiting inflammation. Histology of the murine vaginal mucosa at 2 weeks post-infection of a β-oestradiol treated *C. albicans*-infected mouse (right). The haematoxylin & eosin staining shows a pustule infiltrated by inflammatory cells within the epithelial layer. Immune cells (likely neutrophils) can be seen migrating through the tissue to the site of the pustule. GM-CSF, granulocyte-macrophage colony-stimulating factor; TNF, tumour necrosis factor.

Here, we review the cytokine communication networks that drive the immunopathogenesis of VVC. We summarize how the epithelium maintains an immunological activation threshold to foster asymptomatic vaginal *Candida* colonization. We discuss how the interplay between *Candida* species and the vaginal mucosa catalyses the initial cytokine signalling, and highlight the pivotal components of the innate immune system that have been associated with the dysregulated direction of the inflammatory response during VVC (Fig. 1).

## Keep those fire doors shut: an epithelial tolerance threshold fostering asymptomatic *Candida* colonization

Yeasts of the *Candida* species can be isolated from the vaginal tracts in 20–30% of healthy women, as ‘harmless’ members of their regular vaginal microflora [21]. Although the species *C. albicans* causes almost 90% of VVC infections, it is also persistently found to colonize asymptomatic women [21–23]. Other frequent species isolated from asymptomatic women include *C. glabrata*, *C. tropicalis,* and *C. parapsilosis*, as well as other non-*albicans Candida* (NAC) species [24]. Being opportunistic pathogens, *Candida* species must foster a commensal relationship with the vaginal mucosa by regulating their pathogenicity. While a lot is known about the commensal state of *Candida* species in other mucosal niches (e.g. the intestine and oral cavity) [25–29], less is known about how the fungus maintains commensalism in the vagina. In this niche, *Candida* species may actively establish their commensal status similar to other niches, as intrinsic differences between isolates from asymptomatic women and VVC patients can be observed—specifically in differential induction of the type I interferon (IFN), integrin, and ferroptosis pathways [30]. The vaginal microbiome contributes to maintaining commensalism. Exemplary, *Lactobacillus* species shape the vaginal environment by the secretion of anti-microbial and pathogenicity-inhibiting compounds [31–34]. Short-chain fatty acids (SCFAs) from lactobacilli can affect fungal pathogenicity [35, 36]. Butyrate, however, was also shown to modulate inflammatory responses [36, 37]. Lactate, produced by all lactobacilli is believed to plays a key role in vaginal acidity [38], and is regarded as a major anti-microbial compound for its ability to modulate pathogenicity [39, 40]. Antibiotics that alter vaginal microbiota are associated with VVC susceptibility [3]. Yet, in contrast to bacterial vaginosis, the pH of the vagina surprisingly does not typically change during VVC [41], suggesting a low vaginal pH cannot protect women from VVC. *Candida* species may actively prevent immune activation. In line with this, *C. albicans* produces immunomodulatory compounds, oxylipins, and prostaglandins, which can modulate essential immune responses and promote cell maturation [42, 43]. Fungal prostaglandins can downregulate proinflammatory Tumour Necrosis Factor (TNF) and chemokine production while upregulating anti-inflammatory interleukin (IL)-10 production [43]. Despite the lack of complete knowledge of the roles of oxylipins and other potential immunomodulatory compounds from *Candida* species, this may conjecture that host immune responses can be modulated by *Candida* species to favour their residence as commensals [44].

Asymptomatic colonization by *Candida* species can only be achieved when the host immune system also tolerates their presence. An activation threshold in epithelial cells was proposed as a mechanism that promotes homeostasis by enhancing tolerance to reduce potential frequency and intensity of inflammation induction [13, 45]. Several studies have investigated interactions between vaginal epithelial cells (VECs) and *Candida* species, and suggest a combination of different epithelial mechanisms that tolerate asymptomatic colonization, prevent erratic immune activation, and improve epithelial resistance to infection.

VECs actively control the *C. albicans* growth [46–49]. Interestingly, such anti-*Candida* activity was attenuated in VECs isolated from RVVC patients [48, 49]. Annexin-A1, a mediator of anti-inflammatory cascade and inhibitor of cell proliferation, was identified as a non-inflammatory static inhibitor of *Candida* growth in human oral epithelial cells [50]. It appears plausible with VECs of healthy women similar mechanisms are at play, which altogether regulate colonization with *Candida* species in a non-inflammatory manner and prevent surpassing an activation threshold.

**Figure 2.**
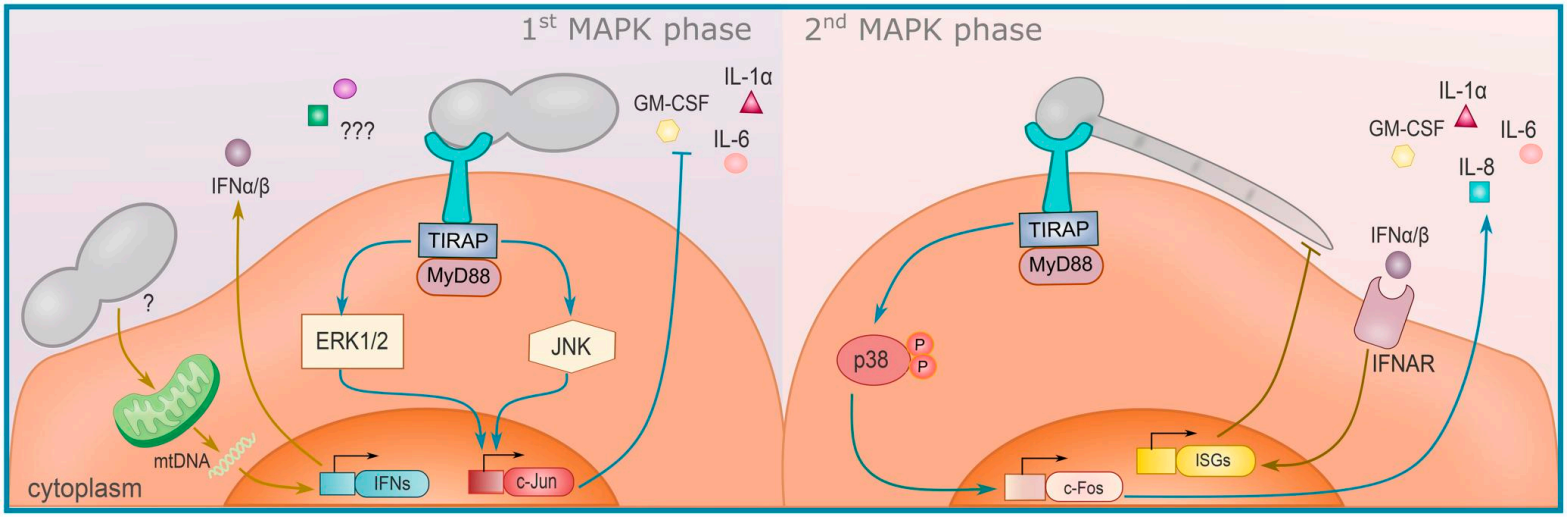
The two-phase mitogen-activated protein kinase (MAPK) and type-I interferon pathways in response to *C. albicans* commensal yeast and invasive hyphae. A closeup from Fig. 1 illustrating the *Candida*-epithelial interactions. *C. albicans* yeast cells activate the first MAPK phase, which is c-Jun-mediated and does not trigger pro-inflammatory cytokines (GM-CSF, IL-1α and IL-6) while promoting tolerance and homeostasis. Yet, specific immune mediators of tolerogenic and homeostatic responses have not yet been identified. Furthermore, *Candida* species can induce a mitochondrial-driven type-I IFN response (left). The second prolonged MAPK phase in parallel with c-Fos transcription factor is only activated when a hyphal fungal burden exceeds a particular level, inducing the production and release of various proinflammatory cytokines. Type-I interferons can bind the IFNAR receptor, triggering the expression of interferon-stimulated genes (ISGs) that mediate epithelial resistance to infection (right). ERK1/2, extracellular signal-regulated protein kinases 1 and 2; GM-CSF, granulocyte-macrophage colony stimulating factor; IL, interleukin; JNK, c-Jun N-terminal kinase; MyD88, myeloid differentiation protein 88; TIRAP, Toll-interleukin 1 receptor (TIR) domain-containing adapter protein.

Interestingly, a certain level of fungal burden, specifically in the invasive hyphal morphology, was shown to be required to trigger downstream immune responses by epithelial cells [51]. Mitogen-activated protein kinase (MAPK) pathways differentiate the response to *C. albicans* hyphae or yeast, in both oral and vaginal epithelial cells [52, 53]. In the presence of fungal cell wall components, such as β-glucan and chitin, nuclear factor-κB (NF-κB) signalling and a first MAPK phase are activated independently of fungal morphology, inducing c-Jun transcription factor production via extracellular signal-regulated kinase 1/2 (ERK1/2) and c-Jun N-terminal kinase (JNK) signalling [52–54]. Only when the hyphal fungal burden exceeds a particular level, the second prolonged MAPK phase is activated together with c-Fos transcription factor (Fig. 2). This induces the production and release of proinflammatory cytokines, such as IL-1α, IL-6, IL-8, and granulocyte-macrophage colony stimulating factor (GM-CSF) by VECs [52]. On oral epithelial cells, only *C. albicans* and *C. dubliniensis* were capable of inducing hyphae and the MAPK/c-Fos pathway [54]. This may directly connect to the capacity of these strains to produce the hyphae-associated toxin candidalysin [55]. While *C. tropicalis* has candidalysin, it did not form hyphae, which is essential for adequate expression of the toxin [54]. Collectively, these studies underscore a conserved MAPK signalling pathway between different epithelial niches, whose second phase is triggered by both, the presence of hyphae and a significant fungal burden. In contrast, the yeast morphotype is tolerated without priming a pro-inflammatory response. Accordingly, a clinical study revealed that symptomatic patients and asymptomatic pseudohyphae/hyphae carriers exhibit a higher expression of Toll-like-receptor (TLR)2, TLR4, EphA2, activation of NF-κB, c-Fos, and p-38 compared to healthy women and asymptomatic yeast *C. albicans* carriers [56].

In parallel to a biphasic MAPK activation, VECs were also found to mount a dynamic biphasic response to several different *Candida* species. This constitutes of an early and general mitochondria-mediated type I IFN signalling and a later species/pathogenicity-specific damage-driven response [57]. Type I IFN signalling induces expression of interferon-stimulated genes (ISGs) via IFN-α/β receptor (IFNAR) signalling and thereby increases epithelial resistance to infection, while simultaneously restricting neutrophil activation (Fig. 2) [57]. Interestingly, *C. albicans* isolates from VVC patients repress type I IFN signalling in VECs [30]. Furthermore, IFNAR neutralization increased epithelial shedding with the asymptomatic isolate but not with the VVC isolate [30]. A role for type I interferons in maintaining vaginal homeostasis can be supported by the finding that vaginal IFNβ levels are higher in healthy women compared to VVC patients, whereas the opposite was observed for IFNα [58]. As these cytokines both signal through IFNAR, several factors may dictate how this axis impacts VVC, such as differences in the localization and dynamics of the type I IFN response, crosstalk with other pro-inflammatory pathways, or interference by the causative strain.

Several mechanisms react in concert to colonization with *Candida* species to provide a buffer that prevents immune activation and minimizes potential damage incurred from the responses. Yet, when a commensal-to-pathogen shift occurs, pro-inflammatory responses are mounted with efficiency (Fig. 2).

## Firestarter: responses of the vaginal mucosa to infection with *Candida* species

When *Candida* species shift from a commensal to a pathogenic state, they can exceed the vaginal-epithelial activation threshold and promote inflammatory responses underlying VVC. Transcriptional responses to the most common *Candida* species causing VVC revealed that vaginal epithelial responses diverge in a species-dependent manner [57]. Compared to *C. albicans*, both *C. glabrata* and *C. tropicalis* cause low damage to VECs, whereas *C. parapsilosis* stays in yeast morphology without invading or causing epithelial damage [57]. This is in line with the largest prevalence of *C. albicans* as the cause of VVC, with around 75%, followed by *C. glabrata* (16.3%), and *C. parapsilosis* (8.9%) [19, 59]. Concurrently, VECs responded according to the damage phenotype rather than to the species. This was underscored using an avirulent *ece1Δ/Δ* mutant, which deviated from the damage-associated signature induced by wildtype *C. albicans* and induced a response similar to non-damaging *C. parapsilosis* in VECs [57]. Owing to its prevalence [19, 60], activation of inflammatory responses induced by *C. albicans* has been thoroughly investigated. Notably, sensing of *C. albicans* expansion by VECs induces several pro-inflammatory cytokines such as IL-1α and IL-6, the neutrophil chemoattractant IL-8, and the granulocyte and macrophage growth factor GM-CSF [52, 61]. This response also includes alarmins and other proinflammatory cytokines, facilitating intense neutrophil chemotaxis to the vaginal mucosa. Activation of the pattern recognition receptors (PRRs), TLR4, and specific intracellular adhesion molecule-grabbing nonintegrin (SIGN)-R1 in response to *C. albicans* drives the release of VEC-derived S100 alarmins (S100A8 and S100A9), which can among other factors induce considerable neutrophil chemotaxis independent from the T-helper (Th)17-pathway [62–65]. As a subgroup of endogenous damage-associated molecular patterns (DAMPs), S100 alarmins can also be released upon cell death [66].

Inflammatory responses are initiated by a variety of *C. albicans* pathogenicity mechanisms. As an example, candidalysin, the cytolytic toxin encoded by *ECE1*, induces MAPK signalling, which is a ‘danger-response’ pathway in oral epithelial cells to promote neutrophil migration [67], which seems to extend also to vaginal cells [52, 61]. In the context of VVC, candidalysin drives tissue damage, neutrophil recruitment in mice, and induces the release of IL-8, granulocyte-colony stimulating factor (G-CSF), GM-CSF, IL-1α, IL-1β, and IL-6 by VECs [61, 68]. On the contrary, *C. albicans ece1Δ/Δ* is incapable of damaging oral epithelial cells and inducing the epithelial p-MKP1/c-Fos-mediated danger responses driving inflammation [69]. In accord with this, candidalysin neutralization using experimental nanobodies blunted vaginal epithelial IL-1α, IL-8, IFNα, and GM-CSF responses, resulting in reduced downstream neutrophil recruitment and activation [61]. Candidalysin deficient *C. albicans* also elicited less release of the chemoattractant CXC chemokine ligand (CXCL)2 and IL-1α, IL-1β, and S100A8 responses, and neutrophil recruitment in a murine VVC model [68].

Apart from candidalysin, secreted aspartyl proteases (SAPs) represent other important *C. albicans* virulence factors [70]. While Sap2 and Sap6 can attract neutrophil migration directly, they also can drive the production of chemokines, such as CXCL2 and IL-8, by the vaginal epithelium *in vitro* and *in vivo* [71]. Sap1 and Sap2 also promote tissue damage in reconstituted human vaginal epithelium [72]. Sap4 and Sap5 are associated with hyphal induction, and are highly expressed during murine VVC where they can promote neutrophil infiltration, suggesting their indirect link with symptomatic VVC infection [73]. *C. albicans* mutants lacking the combination of *SAP1*, *SAP2,* and *SAP3* exhibited decreased virulence in a murine VVC model [74]. Conversely, a different study showed that *C. albicans* deficient in *SAP4-6* led to less vaginal inflammation, and the importance of Sap5 was highlighted for the reduction of neutrophil recruitment and IL-1β secretion, whereas *SAP1-3* deletion did not impact VVC pathogenesis in this study [75].

### The NLRP3 inflammasome

**Figure 3.**
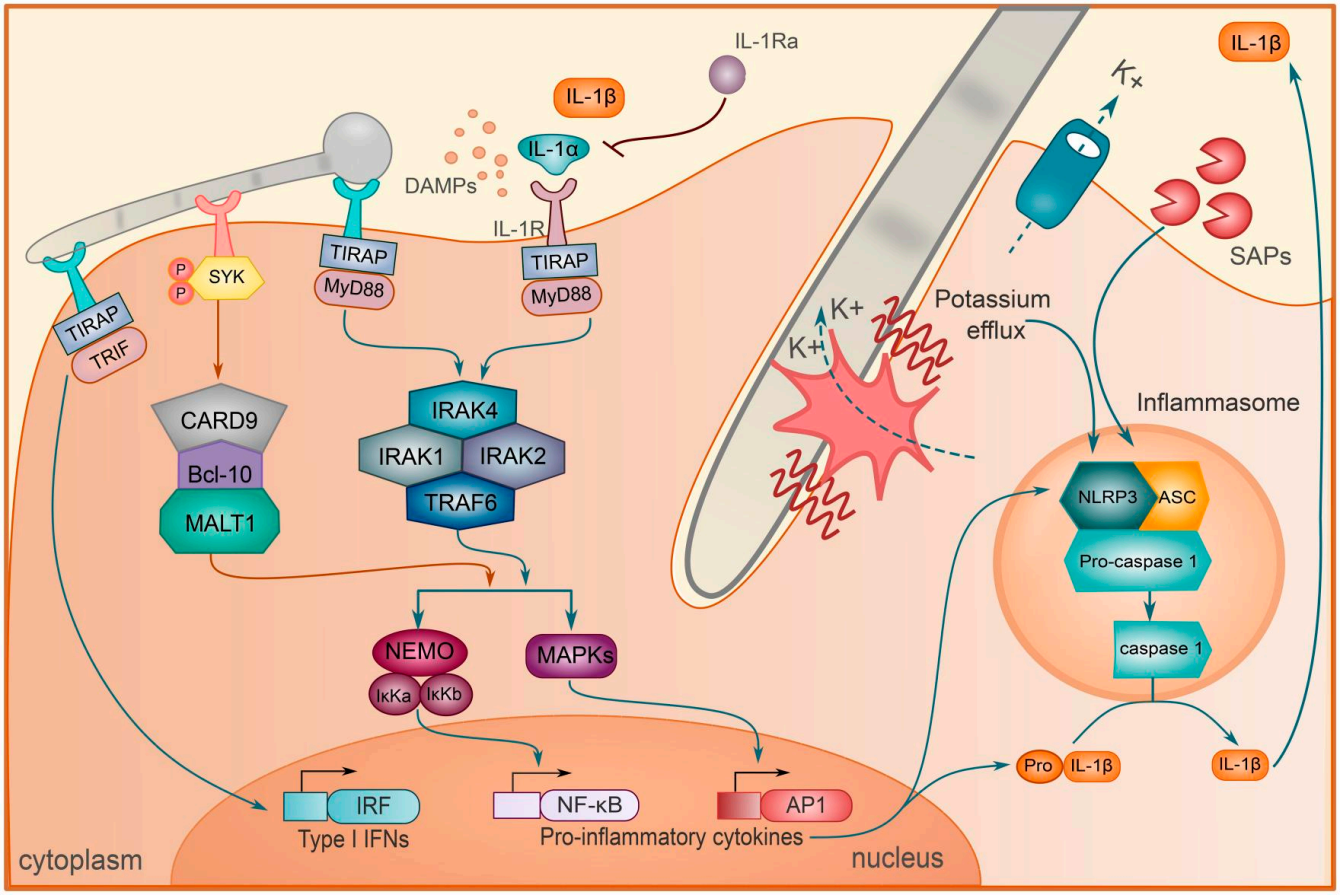
Signalling pathways inducing inflammasome activation during VVC. A closeup from Fig. 1 illustrating several signalling steps converging on IL-1β production in response to *C. albicans*. Recognition of fungal pathogen-associated molecular patterns (PAMPs) by a wide variety of pattern recognition receptors (PRRs), as well as recognition of damage associated molecular patterns (DAMPs) such as the alarmin IL-1α can trigger the activation of a variety of inflammatory transcription factors such as nuclear factor-κB (NF-κB) and activator protein-1 (AP1). These drive the expression of the IL-1β precursor, pro-IL-1β, as well as components of the NOD-, LRR- and pyrin domain-containing protein 3 (NLRP3) inflammasome, also known as inflammasome priming. DAMPs, like extracellular ATP, yet also several *C. albicans* virulence factors such as secreted aspartic proteases (SAPs) and the hyphae-associated toxin candidalysin can activate the inflammasome through a variety of processes, for example by generating potassium efflux. When the NLRP3 inflammasome is activated, pro-caspase-1 is activated to process pro-IL-1β to produce IL-1β. ASC, apoptosis-associated speck-like protein containing a caspase-recruitment domain; Bcl, B-cell lymphoma; CARD, caspase recruitment domain; IFN, interferon; IκKa, IκB kinase α; IκKb, IκB kinase β; IL, interleukin; IRAK, IL-1R-associated kinase; K+, potassium ion; MALT, mucosa associated lymphoid tissue; MAPKs, mitogen-activated protein kinases; MyD88, myeloid differentiation protein 88; NEMO, nuclear factor-κB essential modulator; Pro-IL-1β, Pro-interleukin-1β; SYK, spleen tyrosine kinase; TIRAP, Toll-interleukin 1 receptor (TIR) domain containing adapter protein; TRAF, TNF receptor-associated factor; TRIF, Toll/IL-1 receptor (TIR)-domain-containing adaptor inducing IFN-β.

While the inflammatory response during VVC is initiated by pathogenicity towards the epithelium, the persistence of inflammation and symptoms results from the activation of a protein complex called inflammasome (Fig. 3) [13]. The NOD-, LRR- and pyrin domain-containing protein 3 (NLRP3) inflammasome processes the proinflammatory cytokine IL-1β, a signature cytokine of VVC that contributes to neutrophil recruitment and activation [75, 76]. Inflammasome activation represents a crucial checkpoint in the release of IL-1β by mediating the processing of the inactive pro-IL-1β precursor into its bioactive form, yet IL-1β processing can also occur independent of the inflammasome [77]. Nevertheless, in the context of human VVC, patients can be differentiated from asymptomatic colonized or non-colonized through the expression of *NLRP3* and caspase-1 inflammasome components [78, 79]. Associated with the high inflammasome expression, VVC patients showed elevated IL-1β and IL-8 levels and strong neutrophil recruitment in the vagina [78]. The significance of the inflammasome is mirrored in the murine VVC model, as *Nlrp3* deficient mice show reduced neutrophil recruitment after intravaginal *C. albicans* infection [75]. Recognition of β-glucan, a major component of the cell wall of *Candida* species, by the C-type lectin dectin-1 can lead to activation of the NLRP3 inflammasome in macrophages, and induce a variety of cellular responses leading to TNF, CXCL2, IL-1β, IL-23, IL-6, and IL-10 responses, along with neutrophil recruitment [80–83]. This is noteworthy, as the NLRP3 inflammasome does not only impact IL-1β and IL-18 cytokines processing but also many downstream proinflammatory axes. This results from the shared intracellular signalling domain between TLRs and the IL-1R, which drive a wide range of cytokines under the control of the NF-κB transcription factor [84].

While epithelial cells were shown to mount some inflammasome-dependent IL-1β responses [68, 85], the release of IL-1β by macrophages is an order of magnitude higher [86, 87]. During VVC in mice, macrophages can represent up to 8.3 ± 5.8% of cells in vaginal lavages [88]. Yet vaginal washes and swabs as the most commonly collected samples from patients have the limitation that they do not reflect the cells residing in the tissues. Strikingly, mucosal biopsies of VVC, and particularly RVVC patients exhibited increased presence of CD163 + macrophages [79]. While NLRP3 expression can be visualized throughout the mucosa [79], further research is required to characterize the exact cell types exhibiting inflammasome activation during (R)VVC. Other cells such as monocytes may contribute to the release of IL-1β, given their constitutive active NLRP3 inflammasome [89]. Of note, the chemoattractant MCP-1, which promotes monocytes recruitment, has been found in the murine VVC model [88].

Aside from inducing epithelial MAPK signalling that leads to neutrophil infiltration, candidalysin also contributes to NLRP3 inflammasome activation. In macrophages, candidalysin activates the NLRP3 inflammasome by triggering potassium efflux [76, 86]. Contrastingly, NAC species not expressing candidalysin fail to activate the inflammasome and are impotent in driving both proinflammatory cytokine signalling and neutrophil recruitment [90]. The pore-forming ability of candidalysin also assists *C. albicans* to lyse and escape from macrophages [86, 91, 92]. Macrophage lysis releases a myriad of DAMPs that can further aggravate inflammatory responses [93], yet no systematic investigation looking for DAMPs derived from macrophage lysis during VVC has been conducted.

In addition to candidalysin, SAPs are equally instrumental as inflammasome activators. Sap1 promotes IL-1β release in monocytes *in vitro* [94], and, interestingly, is specifically upregulated in *C. albicans* isolates from VVC patients but not in isolates from healthy women [78, 95]. Sap2 and Sap6 are especially well-characterized inflammasome inducers independent from their enzymatic activity [74, 94, 96, 97]. In particular, Sap2 but also most other SAPs show a higher expression in the vaginal samples from VVC patients [78, 98]. Of note, *C. albicans* Sap2 null mutant was better cleared from the vagina in an oestrogen-dependent rat model [99]. Furthermore, IL-1β release and neutrophil influx could be abolished by treatment with anti-Sap2 immune serum or protease inhibitor, Pepstatin A, or enzyme inhibitor, HuCal I in murine VVC [74]. These highlight the pathogenic role of Saps and their association with the NLRP3 inflammasome in VVC.

## Fight fire with fire: neutrophil-driven immunopathology

Cytokine responses mounted to *C. albicans* infection drive potent neutrophil recruitment. Conventionally, neutrophils can mediate fungal clearance through various effector mechanisms such as phagocytosis, neutrophil extracellular trap (NET) formation, degranulation and release of proteases, oxidative burst, and neutrophil swarming [100, 101]. For this reason, neutrophils are a crucial line of defence during systemic candidiasis [100, 102]. Their recruitment is mediated by several pro-inflammatory cytokines such as IL-1β, IL-8, CXCL1, CXCL2, CXCL5 [68, 78, 103, 104]. IL-17 is a key driver of neutrophil-mediated immunity in many infectious diseases [105] and has been associated with antifungal defence in the oral mucosa. Still, its role in neutrophil recruitment remains unclear given the contradicting observations among serval studies [106–108]. Nonetheless, during (R)VVC, the recruited neutrophils strikingly do not guarantee fungal clearance, but instead seem anergic or even drive symptomatic disease together with the persisting high fungal hyphal burden [16, 20, 109]. Depending on oestrogen administration, changes in the dynamics of neutrophil recruitment and their capacity to clear the fungus were observed [104]. During experimental human vaginal *C. albicans* infection [16], but also in patients [56, 78, 79, 110], vaginal samples from women who developed symptomatic infection showed influx of neutrophils. Impressively, asymptomatic women were not characterized by the clearance of *C. albicans* but rather by the absence of neutrophil influx [16]. This uncoupling of fungal burden from symptomatic *versus* asymptomatic infection rather suggests VVC as an inflammatory disease, which happens to be catalysed by the *Candida* infection [13, 19]. Understanding why infiltrating neutrophils are dysfunctional can help to understand why the vaginal mucosa during VVC can be locked in a chronic inflammatory state that does not resolve the infection.

### Fungal adaptations compromising neutrophil function

Neutrophils and macrophages synergistically mediate the efficient phagocytosis of *C. albicans* [111]. However, fungal adaptations specific to the vaginal environment may impair this clearance. Adaptations in response to oestrogen, enable *C. albicans* to accumulate complement factor H on its surface and evade phagocytosis by neutrophils [112]. *C. albicans* isolates from RVVC patients were found to less potently instigate neutrophil effector mechanisms, and show enhanced levels of reduced thiol that increases their capacity to cope with oxidative stress [113], a key effector mechanism for fungal clearance. How RVVC isolates acquire this resistance remains unclear, but it is possible that the persistence over repeated exposure to neutrophil-mediated inflammation has driven this adaptation. Lactate, an organic acid that dominates in the vaginal environment, and whose concentrations remain high in VVC patients [114, 115], can reduce β-glucan exposure and consequently impact neutrophil recruitment and phagocytosis by macrophages [39]. Further, environmental cues like hypoxia can also similarly reduce β-glucan exposure [116]. The reduced exposure of immunogenic cell wall components and opsonization collectively could compromise fungal recognition and clearance. Nonetheless, in the context of the vaginal environment, it remains unclear whether these processes promote similar immune evasion through β-glucan masking and factor H binding, as unmasking of β-glucan was observed in vaginal isolates from symptomatic patients [117]. In view of this, an in-depth investigation of *C. albicans* behaviour in the vaginal niche in comparison to other niches may help to pinpoint specific fungal adaptations in the vaginal environment associated with neutrophil dysfunction.

### The vaginal environment compromising neutrophil function

On the host side, the vaginal environment has been postulated to promote neutrophil dysfunction, called an ‘anergic state’ [20]. Differences in mice susceptible (C3H/HeN-C57BL/6) or resistant (CD-1) to chronic VVC have revealed intriguing insights into this direction [118]. Compared to susceptible mice, CD-1 mice exhibited less fungal burden over 6 days of the infection, but similar neutrophil recruitment, IL-1β and alarmin S100A8 levels, with a declining but not significant trend over the infection [118]. This underscores that the functionality of the recruited neutrophils is key, rather than neutrophil recruitment *per se*. Heparan sulphate, a ubiquitously expressed proteoglycan on mammalian cell surfaces [119], was proposed to be a key mediator of the neutrophil anergy during VVC [118]. Heparan sulphate was suggested to bind to neutrophil CD11b (MAC1, CR3), and interfere with the recognition of fungal ligands, induction of ROS production, and clearance of *C. albicans* [118, 120]. The correlation between oestrogen levels and the occurrence of VVC may relate to the fact that oestrogens affect heparan sulphate proteoglycan expression [121]. Heparinase III, an enzyme that cleaves heparan sulphate, can rescue neutrophil activity in vaginal conditioned medium from multiple VVC-susceptible (CVVC-S) strains of mice [118]. In this context, a defective NET-mediated antifungal activity associated with chronic VVC was demonstrated in a mouse VVC model, and heparan sulphate was identified as a NETosis inhibitor [122]. Based on these preclinical findings, it would be warranted to investigate whether asymptomatic and VVC patients show differences in heparan sulphate levels. Overall, these studies suggest a dysfunctional state of the recruited neutrophil that fails to effectively deploy its antimicrobial effector mechanisms to eradicate the infection.

### Neutrophil hyperactivation during VVC

Considering the vast antimicrobial arsenal neutrophils carry to the vaginal mucosa, they have a considerable potential to damage vaginal tissues and aggravate VVC. During infection, the myriad of expressed fungal virulence proteins can activate neutrophils. *C. albicans* expresses the zinc-binding molecule, Pra1, which by itself is a potent chemoattractant to neutrophils causing neutrophil influx [103], and can also activate neutrophils directly via CD11b, the α subunit of CR3 [120]. Neutrophils deploy rapid NET formation, mediated through ERK phosphorylation, in response to *C. albicans* hyphae after β-glucan recognition [123]. In line with the observation that vaginal isolates from symptomatic patients show β-glucan unmasking, a strong association with neutrophil levels and extracellular DNA was found [117]. SAPs and candidalysin, which are abundantly expressed during symptomatic disease [78], have been associated with triggering NET formation respectively [124, 125]. In line with this, another study showed elevated markers of NETs such as extracellular DNA with neutrophil elastase and citrullinated histones in vaginal discharge of patients with *C. albicans* vaginitis [126]. While NET formation is otherwise crucial to target large pathogenic *C. albicans* structures like hyphae [127], there is extensive evidence that NETs also mediate host tissue damage [128]. Neutrophils express many proteases that, upon release, also target a wide variety of host proteins thereby causing tissue damage [129]. Associated with the increased marks of NETosis neutrophil elastase was found elevated [126]. Matrix metalloproteinase-8 (MMP-8) is produced by neutrophils at high levels [130] and was found significantly elevated in vaginal washes of VVC patients [115]. To our knowledge, the effects of MMP8 on *Candida* species have not been resolved. Yet, MMP8 may play a key role in VVC immunopathology due to the broad range of host proteins that it can degrade. Upon activation, neutrophils efficiently produce reactive oxygen species (ROS) such as superoxide and hydrogen peroxide [101]. While correlating with the efficiency of *C. albicans* killing [131, 132], ROS can cause dramatic injury to host tissues [133]. Perinuclear anti-neutrophil cytoplasmic antibodies (pANCA), a hallmark of autoimmune vasculitis [134], were found significantly increased in vaginal samples from women with VVC [110]. While these antibodies caused a dramatic release of ROS, this was associated with compromised fungal clearance [110]. The inaccurate release of ROS, due to pANCA for instance, can crucially contribute to tissue damage [135]. This could suggest a hyperactivation of neutrophil extracellular trap formation and ROS release is associated with VVC in humans. Myeloperoxidase, which is normally essential for systemic host defence against *C. albicans* [136], can also inflict damage to host tissues through the oxidation of host molecules by catalysing the production of hypochlorous acid (HOCl) [137, 138]. Collectively, these studies sketch an image that conventional neutrophil effector mechanisms could somehow fail to clear the *Candida* infection during VVC and may worsen the condition. Noteworthy, processes such as ROS release, degranulation, and NETosis, are only effective when they are deployed at the right moment. It is tempting to speculate that the high expression of a variety of secreted fungal effectors (SAPs, candidalysin, and Pra1 [68, 73, 78, 95, 98, 103, 110, 120, 125], as well as host factors such as pANCA [110]) may lead to premature deployment of these effector mechanisms before the neutrophil is in a close enough vicinity for its effectors to harm the fungus.

### Non-self-limiting inflammation

The inflammatory response during VVC has a non-self-limiting phenotype. Many of the proinflammatory cytokines released during VVC can drive positive feedback loops. Exemplary, S100 alarmins recruit neutrophils to the infected vaginal mucosa [62], however, S100A8/A9 is also present in the induced NETs [139], and may thereby promote additional neutrophil recruitment. IL-1β, a hallmark cytokine of the hyperinflammatory response during VVC, drives the recruitment and activation of neutrophils [140]. Importantly, it also potently induces its own production [141]. Normally, IL-1R2 serves as a decoy receptor [142], and IL-1 receptor antagonist competes with IL-1β for IL-1R1 [143]. The self-propagating inflammation during VVC suggests a failed regulation by such endogenous inhibitors, which is exacerbated by activated neutrophils contributing to tissue damage and release of DAMPs that will drive further neutrophil recruitment [144].

Overall, two neutrophil phenotypes have been observed during VVC. Neutrophils can show an inactive ‘anergic’ phenotype which may relate to changes of both the fungi as well as neutrophils in the vaginal environment [110, 118], whereas symptomatic patients also exhibit an overactivation of neutrophils [16, 19]. These contrasting findings underscore the need to elucidate the exact mechanisms of the immunopathogenesis of VVC. Patient stratification can potentially identify groups of patients with inactive and hyperactive neutrophils and identify potential disease subtypes, which may require very different treatment strategies. Such differences in neutrophil functionality may rely on common genetic variation or inter-individual differences in the vaginal niche. Therefore, large cohort studies may be able to shed light on this variation and crucial factors driving it.

## Fire extinguisher or fuel? Adaptive immune responses

Robust induction of IL-17 and IL-22 responses can be observed during VVC [108, 145–147]. Overall, during mucosal *C. albicans* infections, IL-17 and IL-22 have been associated with crucial roles in mediating neutrophil recruitment, production of antimicrobial peptides (AMPs) and epithelial regeneration [148–150], yet the importance and roles of these responses during VVC are difficult to interpret due to the variability in results between studies.

Despite their crucial role at other mucosal sites, some evidence suggests redundancy of IL-17 and IL-22 in VVC: mice deficient for these cytokines did not exhibit a significantly more severe VVC phenotype [108]. Further, induction of potent S100 protein-mediated neutrophil recruitment is still observed in IL-22 and IL-17RA deficient mice [63], although IL-17RA deficient mice did show lower neutrophil numbers. Contrastingly, treatment with halofuginone, an inhibitor of Th17 differentiation [151], was found to exacerbate vaginal *C. albicans* infection through the loss of β-defensin2 [145]. A later study verified that halofuginone-mediated Th17 inhibition compromises a protective role [152]. In a different study, IL-17A or IL-17F deficiency was associated with an early increased fungal burden, yet IL-22 deficiency severely compromised resistance to VVC [147]. This was associated with increased S100A8/A9 release and neutrophil recruitment. In line with this, increasing IL-22 by targeting the aryl hydrocarbon receptor was associated with protection against VVC [146]. IL-18 deficiency was found to mimic the phenotype of IL-22 deficient mice, which can be explained by reduced IL-22 levels [146]. However, cells other than traditional T-helper cells can mediate these responses. Deficiency in γδ T-cells was shown to impair resistance to vaginal *C. albicans* infection [153], and even lead to uterine *C. albicans* infection [154]. The protection was associated with mediating IL-17-dependent neutrophil recruitment, however, given the detrimental role of neutrophils in VVC, the protective effect may rather rely on other mechanisms such as γδ T-cells promoting tissue repair or expression of AMPs.

The role of T-cell-mediated responses remains controversial. The discrepancies between studies may be explained by differences in microbiomes of laboratory animals and subtle differences in the experimental system. However, in women, the contribution of T-cell-mediated responses could be a group-dependent occurrence. In this context, genetic background may play a decisive role as common variations in *IL23, IL17A* and *IL17F* genes are associated with elevated cytokine serum levels and increased risk of RVVC in the Chinese ethnicity [155]. The NDV-3 vaccine, which was in clinical trials for VVC prevention [156], was shown to induce humoral, IFNγ and IL-17 responses. Further, NDV-3 relies on T-cells to mediate its protection against VVC in mice [157]. This underscores that protective immunity against VVC can be achieved via adaptive responses.

## An overall fire hazard: altered systemic immune susceptibility to RVVC

Even though VVC is a local infection of the vaginal mucosa, healthy women and RVVC patients exhibit differences in the responses by systemically circulating immune cells. Circulating peripheral blood mononuclear cells (PBMCs) from RVVC patients showed significantly higher responsiveness to *C. albicans* hyphae in terms of TNF release [158]. Further RVVC patients showed an altered balance in the ratio between anti-inflammatory cytokines like IL-10 and IL-1Ra, and TNF and IL-1β respectively. Interestingly, this was associated with atopy reported in the RVVC patients [158]. A family history of atopy and eczema is associated with a poor response to fluconazole maintenance therapy for RVVC [159]. In this context, the IL-9-mast cell axis, which promotes allergic inflammation in other niches was shown to drive early IL-1β responses [160], yet later IL-1Ra mediated resolution of inflammation in a murine VVC model [161]. Intriguingly, RVVC patients show a trend towards elevated vaginal IL-9 levels, which were associated with high IL-1β and low IL-1Ra levels [161]. In line with the hypothetic allergic hypersensitivity phenotype associated with RVVC, a common genetic variation in the promotor region of the allergy-associated cytokine IL-4 was related with disease [162]. Patients with RVVC also show higher vaginal IL-4 levels, which may relate to the fact that the polymorphism was found to regulate IL-4 release [162].

Interestingly, common genetic variations in crucial immune genes are enriched in RVVC patients and associated with their altered susceptibility to vaginal *C. albicans*-mediated infections as well as differences in systemic immune responses [163]. A common variation in TLR2 was associated with a 3-fold increase in susceptibility to RVVC, and women with this variation show impaired IL-17 and IFNγ responses to *C. albicans* [164]. However, whether these impaired Th cytokine responses directly underly the increased susceptibility remains elusive. Variation in the sialic-acid receptor SIGLEC15 associates with increased risk of RVVC, and women carrying at least 1 mutated allele showed enhanced IL-17, IL-22, and IFNγ release. Yet, the polymorphism also associates with increased NLRP3 and IL-1β expression, which was also observed in mice upon *SIGLEC15* silencing [165]. Variable number tandem repeats in *NLRP3* have also been associated with RVVC [166, 167]. While some of these variations were associated with an increased *NLRP3* expression and IL-1β processing, another association was made with a variant leading to decreased NLRP3 production and thus IL-1β processing [167]. This may suggest that susceptibility to RVVC can potentially underly both hyperactive as well as poor responsive IL-1β processing and signalling. In line with this, polymorphisms compromising dectin-1 function and thus mounting a potent response to fungi have been associated with VVC [168]. Similarly, variation in *MBL*, a soluble c-type lectin has been associated with RVVC susceptibility in various studies [169–173].

Even though altered responses of systemically circulating PBMCs have been observed in RVVC patients in terms of genetic variation and allergic phenotypes [158, 164, 165, 168], these changes may also reflect the extent of how mucosal immunity and cytokine signalling network during RVVC is affected. As mucosal immunity is challenging to assess using non-invasive techniques, changes in responses of systemically circulating immune cells may be able to reflect dysfunctional immunity to *Candida* species predisposing to RVVC.

## Future perspectives

### Unexplored inflammatory axis in VVC pathogenesis

#### Is there a role for the extended IL-1 family in VVC?

The role of IL-1β and the NLRP3 inflammasome in VVC has been very well studied [75, 90, 98]. Still, it is not fully understood why the inflammasome is so strongly activated during VVC. However, particular characteristics of the vaginal environment may be involved. In contrast to IL-1β, IL-1α can be released as a DAMP by damaged epithelial cells [61]. Both cytokines signal through IL-1R1 and its accessory protein IL-1R3, which can be efficiently inhibited by IL-1Ra that competitively binds IL-1R1 but does not recruit the accessory protein [174]. In accord with this, IL-1Ra deficient mice show an increased susceptibility to VVC and IL-1Ra administration was found to limit VVC pathogenesis [175]. Despite being a potent endogenous antagonist of VVC-pathogenesis driving IL-1 signalling, IL-1Ra has not been explored in-depth in VVC patients. Based on the compelling evidence provided from mouse models and a group of patients [175], it is plausible that a failure of endogenous IL-1Ra-mediated suppression to IL-1 signalling could be a susceptibility mechanism. Interestingly, IL-1Ra may also contribute to the activation threshold of VECs as it can be induced by lactobacilli when the microbiome is intact [176], as well as their metabolite lactic-acid [177]. A compromised *Lactobacillus*-dominated vaginal microbiome may thus lower the activation threshold by removing the release of IL-1Ra. A systematic analysis of IL-1Ra levels in (R)VVC patients and asymptomatically colonized women may shed more light on the role of this cytokine in VVC.

Apart from IL-1α, IL-1β, and IL-1Ra, the IL-1 family consists of 7 additional members, each having specific receptors, inhibitors, antagonists, or anti-inflammatory functions [77]. Many of the other IL-1 family cytokines have been reported to play specific roles in antifungal host defence [178]. The IL-36 subfamily exerts proinflammatory characteristics similar to the IL-1 subfamily in driving neutrophil responses [179]. Particularly, IL-36 cytokines support *C. albicans*-induced Th17 responses [180, 181]. Thereby, these cytokines have been implicated in the immune response during oral candidiasis and *C. albicans* keratitis [181–183]. IL-36 signalling can be antagonized by both its natural IL-36 receptor antagonist (IL-36Ra), but also the anti-inflammatory IL-1 family cytokine IL-38 [180]. Given the similarity in biology to IL-1, it appears conceivable that cytokines of the IL-36 subfamily as well as IL-36Ra and IL-38 may also play a role in the immune response during VVC [179].

IL-18 is a member of the IL-1 family, best known for its capacity to drive IFNγ and Th1 responses during *C. albicans* infections. Even though IL-18 is also processed by the NLRP3 inflammasome during murine VVC [74], it was not found elevated in VVC patients [166]. Yet, IL-18 deficient mice show an increased susceptibility to VVC [146]. Exploring the IL-18 axis in further depth may reveal a dichotomy of NLRP3 activation with IL-1β driving detrimental inflammation effects whereas IL-18 mediating protective effects. Of note, IL-18 can context-dependent also promote type 2 immunity and can act directly on basophils and mast cells to release IL-4, IL-13 and histamine, particularly in absence of IL-12, rendering IL-18 a potential driver of atopy in specific VVC patient subgroups [184–186]. Within the IL-18 subfamily, IL-37 represents an anti-inflammatory cytokine signalling through IL-18Rα and SIGIRR (IL-1R8) [187]. The absence of IL-37 in mice complicates studying the role of this cytokine in VVC using the mouse model [187]. However, when transgenically expressed in mice, human IL-37 triggers a functional pathway, which potently inhibits neutrophil recruitment in the context of peritoneal *C. albicans* infection [188]. In an aspergillosis model, IL-37 was found to inhibit NLRP3 inflammasome activation [189]. In the context of VVC, these anti-inflammatory effects mediated by IL-37 could be relevant in preventing immunopathology. Interestingly, IL-37 is expressed in VECs infected with different *Candida* species [57]. Thus, it could be a promising avenue to explore whether a failure of IL-37 to control hyperinflammation contributes to VVC pathogenesis. Finally, IL-33, prominent in allergic inflammation, is another poorly studied cytokine in VVC. IL-33 is broadly expressed in epithelial barriers including stratified squamous vaginal epithelium, is commonly released by tissue damage and can be matured to enhanced activity by foreign proteases [190, 191], like it was shown in *A. fumigatus*, for instance [192]. As *C. albicans* has an expanded protease arsenal and a vaginal papain protease allergy model drives inflammation via IL-33 [193], it appears quite possible that IL-33-driven inflammation might be enhanced by fungal proteases during VVC. IL-33 can directly activate all flavours of granulocytes and prime recruitment and antifungal capacity of neutrophils [186, 194–196], thereby potentially promoting protection against systemic candidiasis. This could partially be explained by IL-33-boosted upregulation of granulocyte surface CD11b and thereby enhanced antifungal effector functions including phagocytosis [194]. Beyond that, IL-33 promotes Th2 cell activation [197, 198], making it a potential target to intervene with atopy in subgroups of RVVC patients.

#### Eicosanoids

Lipid mediator eicosanoids such as leukotrienes and prostaglandins crucially mediate acute inflammatory responses and are formed from arachidonic acid [199]. Leukotriene B_4_ is known as a potent neutrophil chemoattractant and may play a role during VVC. Interestingly, specifically *C. albicans* hyphae, which are associated with symptomatic VVC, can potently induce the production of leukotriene B_4_ in human neutrophils and macrophages [200, 201]. However, in a murine VVC model leukotrienes were found dispensable [202]. Studies using VVC patient cohorts could help to identify whether eicosanoids play a role during the acute inflammatory response.

#### Cytokines contributing to the immune activation threshold

Tolerogenic responses to *Candida* species are instrumental in maintaining the threshold of the immune system not to respond to commensal colonization. IL-34 is a constitutively expressed cytokine by skin keratinocytes that promotes macrophage polarization towards the M2 subtype, known as anti-inflammatory or alternatively-activated macrophages, which are mostly involved in tissue repair [203]. IL-34 was found to significantly reduce TNF release by M1 macrophages in response to heat-killed *C. albicans*. Furthermore, an IL-34-mediated response was associated with suppression of TLR2 and dectin-1 expression, which recognize α-1–4-glucans, and β-1,3-glucans respectively*,* in M1 macrophages [204, 205]. Despite its instrumental role in macrophage polarization and tissue homeostasis, IL-34 has not been explored in the context of VVC. Characterizing molecular pathways regulated by IL-34 could shed light on its role in responding to and tolerizing *C. albicans* pathogenicity in the vaginal mucosa.

### VVC ≠ VVC

Even though VVC is classified as one disease, a wide range of the symptomatology of VVC can be observed and is linked with distinct pathogenicity of different *Candida* species [206]. Most of our knowledge of the pathogenesis, particularly the immunopathogenesis, of VVC is based on *C. albicans*. However, milder symptoms are generally observed in women infected by NAC species [207–209]. This may pertain to the comparatively low level of tissue damage, and thus release of cytokines, these species cause, which is linked to their capability in hyphae formation [57]. Five out of the six most representative NAC species—*C. tropicalis*, *C. parapsilosis*, *C. krusei*, *C. glabrata*, and *C. auris*, except *C. dubliniensis*, were found to neither form hyphae in the mouse vagina nor activate the NLRP3 inflammasome; ultimately, no vaginal immunopathology is elicited [90]. Even though *C. dubliniensis* can switch its morphology, its low filamentation rate diminishes its adhesion, colonization, and dissemination in the host, accompanied with lower virulence [90, 210, 211]. *C. tropicalis* also can form hyphae, but it shows lower *ECE1* expression [55, 90]. Specifically, *C. glabrata* lacks most of the virulence factors (candidalysin, Pra1, SAPs) that were identified in *C. albicans* to play a role in VVC pathogenesis. In line with this, *C. glabrata* caused murine infection with markedly low induction of IL-1β and S100A8 responses and consequent neutrophil recruitment [212]. This could suggest that the infection with NAC species rather relies on the fungal pathogenicity mechanisms than the inflammatory response. Species like *C. glabrata* that seem otherwise avirulent in *in vitro* models, can still infect and inflict damage to vaginal epithelial cells when human (but not mouse) albumin, an abundant vaginal niche factor, is present [213]. Yet, it remains unknown how *C. glabrata* in this context can induce cytotoxicity in vaginal epithelial cells, while preventing activation of inflammatory responses by released DAMPs. A connection between the induction of neutrophil recruitment and whether strains have the *PRA1* gene has been recently shown in *C. albicans*. Strikingly, *C. glabrata*, which does not have the *PRA1* gene fails to induce neutrophil recruitment, unless it is transgenically introduced [103]. The ambiguity of relatively high incidence of *C. glabrata* in the human population but the lack of apparent pathogenicity or virulence in animal models underscores the need for further investigating VVC caused by this species.

Even among *C. albicans* VVC isolates, a phenomenal heterogeneity in *C. albicans*-macrophage interactions, filamentation capacity, cell adhesion, cell damage, and cell wall architecture are observed [214, 215]. *C. albicans* strains that are defective in Efg1-driven hyphal formation revealed that the yeast-to-hypha switch and its related virulence are compulsory for the induction of strong inflammatory responses during *C. albicans* vaginitis [216]. However, other evidence suggests that at the acidic pH of the vagina rather a pseudohyphal morphology phenotype is observed, which expresses inflammation-inducing factors specific to yeast (SAP2) as well as hyphae (ECE1) [98]. While *ECE1* expression is generally higher in symptomatic VVC patients [78], its expression does not strongly correlate with IL-1β and IL-8 levels [103]. An explanation for this can be the existence of different *ECE1* allele variants, which result in reduced candidalysin generation, and in consequence profoundly reduced IL-1β secretion, neutrophil recruitment, and tissue damage in vaginal lavage [217]. Comparison between VVC and RVVC isolates revealed increased SAP activity in RVVC isolates, which was also associated with a stronger capacity to elicit cytokine responses [218]. The heterogeneity among the disease presentation between (R)VVC patients may thus strongly rely on the *Candida* strain causing the infection. However, the presence of other microbes can also drastically impact immune responses. *Streptococcus agalactiae*, a common colonizer of the vaginal mucosa aggravates IL-1β, responses when co-infected with *C. albicans* [219]. *Lactobacillus crispatus* can modulate VEC innate response to *C. albicans*. A drastic increase of proinflammatory cytokine IL-6 can be observed, stimulating the host’s defence against pathogens [220]. As discussed before, inter-individual variability between women can be determining a different disease manifestation, specifically, the presence of certain genetic variations conferring susceptibility [163]. Individual differences in microbiome, behavioural or hygiene practices may also potentially associate with a different disease phenotype [221]. Hence, patient stratification may help to identify specific subclasses of VVC that each would benefit from different treatments, such as pathogenicity suppressing treatments or inflammation suppressing treatments. However, also systemic variation in the immune system represents an important variable dictating disease phenotype.

## Conclusions

To date, characteristics of VVC and the involved components have been detailed by numerous animal and patient studies. This helps reclarify the immunopathological nature of VVC and highlight the importance of several major mechanisms. An epithelial innate immune activation threshold protects against unnecessary activation of the immune response to *Candida* colonization. During infection the epithelial activation threshold is overcome and will lead to cytokine signalling. In parallel, the NLRP3 inflammasome drives IL-1β-mediated inflammation and neutrophil recruitment. During VVC, the recruited neutrophils worsen the condition as they are dysfunctional within the vaginal niche and fail to clear the infection. Several cytokine axes have so far been unexplored in VVC and their investigation can help to get a comprehensive understanding of VVC immunopathogenesis. Particularly anti-inflammatory cytokines that protect against inflammatory pathology may represent a promising direction to uncover therapeutic targets.

The continuously improving understanding of these mechanisms in VVC has changed our thinking that VVC is rather an inflammatory disease than just an infection. Specifically, this may impact how VVC is diagnosed and treated. Furthermore, the participation of numerous factors—vaginal environment, vaginal microbiota, *Candida* species and strain, the host genetics, and the immune status—in influencing inflammatory responses in VVC has been demonstrated. This could call for stratification of VVC into multiple subtypes, where each subtype should be investigated individually rather than generally, to facilitate personalized approaches in VVC treatment.

## Authors’ contributions

Kar On Cheng (Investigation [lead], Writing—original draft [equal], Writing—review & editing [equal]), Dolly Estella Montaño Espinosa (Data curation [equal], Investigation [supporting], Supervision [supporting], Visualization [lead], Writing—review & editing [equal]), Teresa Zelante (Visualization [supporting], Writing—review & editing [equal]), Axel Dietschmann (Data curation [equal], Funding acquisition [supporting], Investigation [supporting], Supervision [supporting], Writing—review & editing [equal]), and Mark Gresnigt (Conceptualization [lead], Funding acquisition [lead], Investigation [supporting], Project administration [lead], Supervision [lead], Writing—original draft [equal], Writing—review & editing [equal])
